# Assessment on Inpatient Glycaemic Control in General Medical Wards, Putrajaya Hospital

**DOI:** 10.21315/mjms2024.31.2.18

**Published:** 2024-04-23

**Authors:** Nor Nadziroh Ibrahim, Nurain Mohd Noor, Rashidah Bahari, Lisa Mohamed Nor, Nurul Huda Zainal Abidin

**Affiliations:** Clinical Research Centre, Hospital Putrajaya, Wilayah Persekutuan Putrajaya, Malaysia

**Keywords:** type 2 diabetes mellitus, stress hyperglycaemia, prevalence, glycaemic control

## Abstract

**Background:**

Inpatient hyperglycaemia is common and associated with poor outcomes such as increased mortality and prolonged hospital stay. This study aimed to determine the prevalence of inpatient hyperglycaemia and glycaemic control in Putrajaya Hospital, Malaysia. Secondary objectives were to compare the length of stay (LOS), 30-day readmission rate, and death between controlled and uncontrolled glycaemic groups.

**Methods:**

This cross-sectional study was conducted between 1 July and 31 December 2019 among patients in medical wards who had a blood glucose (BG) level of > 7.8 mmol/L and stayed in the wards for ≥ 24 h. We retrieved information on demographics, diabetes history and BG profiles. The definition of controlled glycaemic status is when ≥ 80% of BG readings were between 4.0 mmol/L and 10.0 mmol/L during the hospital stay.

**Results:**

The prevalence of inpatient hyperglycaemia was 55.2%. There were 841 patients who met the eligibility criteria; their mean age was 60 (13.8) years old. Most (79.4%) of the patients were Malay and 53.9% were male. There were 452 (53.7%) patients in the uncontrolled group. They were younger and admitted with more kidney complications compared to those in the controlled group. The median LOS for both groups was 3 (2) days. The uncontrolled group showed a higher percentage of readmission within 30 days (7.5% versus 4.6 %) and death during admission (3.3% versus 1.6 %) (*P* = 0.100 and *P* = 0.082).

**Conclusion:**

The prevalence of inpatient hyperglycaemia was high. More than half of them had uncontrolled BG. Both groups had a similar average length of stay. The 30-day readmission rate and death during admission were higher in the uncontrolled group, although statistically not significant.

## Introduction

In Malaysia, type 2 diabetes mellitus (T2DM) is a serious public health concern due to the high rate of complications and mortality. The prevalence of T2DM is increasing worldwide. The National Health and Morbidity Survey (NHMS) 2019 reported that diabetes prevalence among adults aged 18 years old and above has increased to 18.3% from 17.5% in 2015 (1).

Patients with T2DM have a greater chance of hospitalisation than those without the disease. They are hospitalised for diabetes-related complications and stay longer in the hospital (2). In the United Kingdom, the 2016 National Diabetes Inpatient Audit (NaDIA) suggested that the prevalence of diabetes amongst inpatients had risen from 15% in 2010 to 17% in 2016 (3). While in the United States, approximately 22% of all hospital inpatient days were incurred by people with diabetes (4). Another study in Tübingen University Hospital, Germany, whereby 3,733 adult patients were screened for diabetes and prediabetes over 4 weeks, found that almost every fourth hospital patient (22%) had diabetes (5).

Inpatient hyperglycaemia is a term describing inpatients with diabetes and stress hyperglycaemia (SH). The prevalence of inpatient hyperglycaemia in various studies ranged from 38% to 45% (6–9). Inpatient hyperglycaemia is linked to negative outcomes such as increased mortality and duration of hospital stay (10, 11). Hyperglycaemia during acute illness may reflect either pre-existing diabetes (known or undiagnosed) or SH. SH is a transient increase in blood glucose (BG) in the absence of diabetes that occurs under acute physiological stress and resolves spontaneously when the acute illness has subsided. An acute illness can exacerbate hyperglycaemia through elevations in stress-related hormones (growth hormone, catecholamines, cortisol and glucagon), pharmacologic agents (steroids, anti-psychotics and inotropes), enteral and total parenteral nutrition (12). Hyperglycaemia, in turn, triggers physiological changes that exacerbate the acute illness, such as decreased immune function and increased oxidative stress. This leads to a vicious cycle of worsening illness and poor glycaemic control. Thus, proper management of hyperglycaemia is crucial.

Glucose monitoring is essential during hospitalisation to guide the treatment regime and prevent hypoglycaemia or hyperglycaemia. The American Diabetes Association (ADA) recommends bedside BG to be checked before meals in patients taking meals, patients who are not eating should have their glucose levels checked every 2 h–4 h, and patients who receive intravenous insulin require more frequent glucose monitoring, ranging from every 30 min to every 2 h (11).

According to the Practical Guide to Inpatient Glycaemic Care, Ministry of Health Malaysia 2020, insulin is the preferred pharmacological therapy for most inpatient hyperglycaemia. It should be initiated when the BG level persistently > 10.0 mmol/L. However, some patients without contraindication to oral anti-diabetic (OAD) usage can continue their home medications while in the hospital. Most non-critically ill patients should have a target plasma glucose of 7.8 mmol/L to 10 mmol/L (12). This recommendation was consistent with ADA; “for the majority of non-critically ill patients treated with insulin, premeal glucose targets should generally be < 7.8 mmol/L in conjunction with random BG values < 10.0 mmol/L, as long as these targets can be safely achieved” (4).

Ables et al. (10) found that 52.3% of patients with hyperglycaemia had good glycaemic control when ≥ 80% of their BG values were between 4.0 mmol/L and 10 mmol/L throughout hospital stay. Moreira et al. (13) sought to assess glycaemic control by grouping patient’s BG readings by calendar day before calculating the mean for each patient day. Overall, they found the percentage of patient days with BG values within a predefined optimal range of 4.4 mmol/L to 7.8 mmol/L was 11.8% in general wards.

In addition to the increasing prevalence of diabetes in the hospital, many patients without pre-existing diabetes experience stress-related hyperglycaemia during hospitalisation. Inpatient hyperglycaemia is, therefore, common and related to an increased risk of complications. Our country lacks evidence on the prevalence of hyperglycaemia and glycaemic control among hospitalised patients. Thus, the objectives of our study were to investigate the prevalence of inpatient hyperglycaemia and to determine glycaemic control among inpatients with hyperglycaemia in Putrajaya Hospital, a community hospital in the federal government administrative centre of Malaysia. We also compared the characteristics, hospital length of stay (LOS), death rate during admission, and 30-day readmission rate in controlled and uncontrolled glycaemic groups.

## Methods

### Study Design and Sample Size

This was a cross-sectional study using retrospective data of patients aged 18 years old or more who were admitted to general medical wards of Putrajaya Hospital during a 6-months period starting from 1 July 2019 until 31 December 2019. Medical records of patients with bedside capillary BG values of more than 7.8 mmol/L during admission were retrieved from the Electronic Medical Record (EMR). A simple random sampling method was used to recruit patients in this study.

Sample size estimations were calculated with a 95% level of confidence and 0.05 precision using the population proportion formulae (14). Prior data indicate that the prevalence of hyperglycaemia amongst inpatients was 0.40 (7) and the proportion of controlled glycaemic control was 0.52 (10). Meanwhile, the hospital mortality death rate in the controlled and uncontrolled groups was 0.7% and 1.19%, respectively (10), while the 30-day readmission rate in the controlled and uncontrolled groups was 14.1% and 16.6%, respectively (10). Considering the additional 20% dropout rate, the largest sample size would be 480. For LOS, sample size estimation was calculated using two population mean formulae (14). Based on prior data, the mean LOS for the controlled and uncontrolled groups were 5.9 ± 1 and 6.2 ± 1 days, respectively. Power was set at 0.8 and the type I error at 0.05. Thus, a minimum sample size of 175 samples per group was required. With an additional 20% dropout rate, the final sample size was 219 samples per group. Therefore, the largest sample size was 480 samples.

### Definition

BG measurement in this study refers to bedside capillary BG measurement using point-of-care glucose meters, as it is currently the recommended method of glucose monitoring in inpatients (11). Hyperglycaemia in hospitalised patients is defined as a BG level > 7.8 mmol/L (11). T2DM was diagnosed based on either a history of T2DM or a BG of > 7.8 mmol/L and elevated HbA1c in patients who had no prior history of T2DM (15). SH is a BG value of > 7.8 mmol/L present during acute illness in patients with previously normal glucose tolerance and no history of diabetes (15). On the same note, elevated glycosylated haemoglobin (HbA1c) of ≥ 6.3% without a history of T2DM aids in distinguishing newly diagnosed T2DM from SH (15). BG readings were defined as controlled if 80% or more of their BG readings during hospitalisation were between 4.0 mmol/L and 10.0 mmol/L, whereas uncontrolled if less than 80% of their BGs fell within this range (10).

Exclusion criteria were: i) patients who required intensive care unit (ICU) admission; ii) admission for diabetic emergency, namely diabetic ketoacidosis (DKA), hyperosmolar hyperglycaemic state (HHS) and hypoglycaemia; iii) pre-existing T1DM; iv) pregnancy; v) steroid usage; vi) advanced chronic kidney disease (CKD) (Stages IV and V) and vii) hospice or palliative care including Ryle’s tube feeding during hospital admission.

### Data Collection

Before collecting data, all study team members had been briefed on the definition and data needed in this study using a standardised case report form. Patients who fulfilled the inclusion and exclusion criteria were identified and their data were further explored. Data regarding diabetes (diagnosis, management and complications), sociodemographics, reason for admission, hospital LOS and outcome (discharge, death, and readmission within 30 days) were documented. For each patient, their BG charts were extracted and analysed before categorising the patient into either the controlled group or the uncontrolled group. Besides that, HbA1c values measured within 3 months before or during hospitalisation were recorded. OAD agent and insulin administrations were documented. All data were rechecked and verified by the Principal Investigator to ensure standardisation and good data quality. These data and information were entered into CRF before inputting into an Excel file for subsequent data analysis.

### Statistical Analysis

The data analyses were performed using the IBM SPSS for Windows version 21.0. First, the data from the Excel file were manually entered into the software. Data cleaning was conducted to detect any errors that could affect the accuracy of the results. Only then the actual analysis was carried out. Descriptive statistics were used for selected variables. The findings were presented according to the types and distribution of the data. Categorical data (gender, race, type of inpatient hyperglycaemia, complications of T2DM, type of medications, reason for admissions and admission outcome) were presented as frequencies (percentages). While numerical data (age, durations of T2DM, fasting blood sugar [FBS], HbA1c and LOSs) were presented as means (standard deviations) (if normally distributed) or in medians (interquartile ranges) (if not normally distributed).

A comparison of the differences in normally distributed numerical data between two independent groups was analysed using the independent *t*-test. In contrast, the Mann-Whitney U test was used if the data were not normally distributed. To study the association between two sets of categorical data, Pearson’s chi-square test for independence was used.

## Results

**Figure 1:**
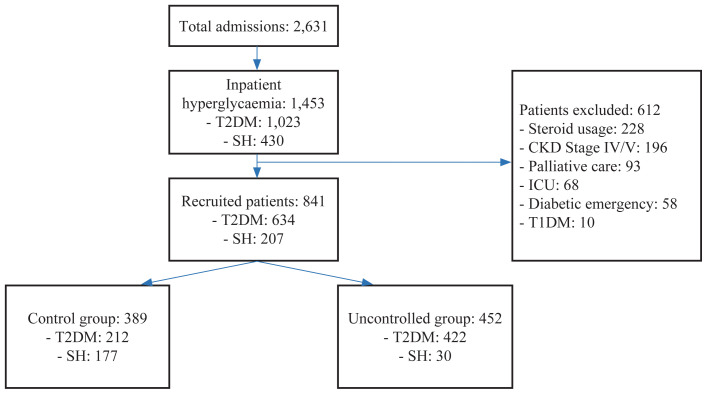
Distributions of study population based on type of hyperglycaemia

The prevalence of inpatient hyperglycaemia in our study was 55.2% (1,453 out of 2,631), of whom 1,023 (38.9%) patients had diabetes and 430 (16.3%) patients had SH (Figure 1). In total, 612 (42.1%) patients with hyperglycaemia had to be excluded from this study due to exclusion criteria, while 841 (57.9%) patients fulfilled the eligibility criteria, with three-quarters having T2DM. When these patients were classified based on their glycaemic control, we found that 452 (53.7%) patients had uncontrolled BG, with most having T2DM. The demographic characteristics of patients in the controlled and uncontrolled groups are summarised in Table 1.

The majority of our study population was represented by Malay males with a mean age of 60 years old. About half of the patients in our study were admitted due to infections such as pneumonia, acute gastroenteritis, urinary tract infection and cellulitis, which became the commonest reason for admission in both groups, followed by cardiovascular diseases (examples were acute coronary syndrome and cardiac failure). When comparing both groups, patients in the uncontrolled group were significantly younger, with most of them having T2DM. On the other hand, controlled group had more SH patients than in uncontrolled group. Nephrology cases (mostly acute kidney injury) were statistically higher in the uncontrolled group than in the controlled group. However, in terms of gender and race, there were no significant differences between the groups.

As given in Table 2, patients in the controlled group had a significantly higher chance of being discharged home than patients in the uncontrolled group. Conversely, the uncontrolled group had more patients who were admitted again within 30 days as well as death during admission, although statistically not significant. As expected, the uncontrolled group had significantly higher fasting blood sugar and HbA1c than the controlled group. In the uncontrolled group, 86.9% of patients required inpatient treatments, mainly with insulin injection, compared to only 33.4% of patients who required treatment in the controlled group.

## Discussion

We discovered that hyperglycaemia was present in 55.2% of inpatients, among which 38.9% had T2DM and 16.3% had stress hyperglycaemia. This prevalence was higher than in China, whereby an observational multicentre study involving three large urban hospitals in Nanjing found that hyperglycaemia among inpatients in internal medicine was 45.7% (8). Compared to our study, both involved Asian countries and included medical patients. However, notable differences in ethnicity were observed, with most patients in our study being Malays. In Argentina, there was a retrospective cohort study done at a tertiary referral hospital involving medical and surgical patients with a median age of 70 years old and equal gender distribution, showing a prevalence of inpatient hyperglycaemia of 40.4% with an incidence of SH of 12.13% (6). Another study in a teaching hospital in Mexico involving medical and surgical patients with a mean age of 52 years old and predominantly males showed the prevalence of inpatient hyperglyacemia was 35%, with 15.6% being afflicted with SH (9). These two studies demonstrated a lower prevalence of inpatient hyperglycemia compared to our study, which could be attributed to variations in ethnic compositions and study populations. Specifically, these studies included medical and surgical patients, whereas our study exclusively focused on medical patients. Overall, the relatively high prevalence of inpatient hyperglycaemia found in our study might be because of the high prevalence of diabetes (diagnosed or undiagnosed) in our country. According to the International Diabetes Federation, the prevalence of diabetes in adults aged 20 years old–79 years old in 2019 in the world was 8.3%. The prevalence in Southeast Asia was 11.3% (16), whereas in Malaysia, the latest NHMS 2019 survey reported higher diabetes prevalence, which was 18.3%, and this percentage has increased from 17.5% recorded in 2015 (1).

When BG values were analysed, over half of our study patients had uncontrolled BG. Ables et al. (10), who used a similar methodology, reported that nearly half (47.7%) of inpatients had uncontrolled BG. The study involved patients 18 years old and older admitted to general medical (including DKA and HHS not treated in ICU), surgical, and psychiatric units. In the study, the mean age was 64 years old in both groups, and the percentage of male and female patients was significantly higher in the controlled group than in the uncontrolled group. The study counted BG starting 48 h after admission, which explained better glycaemic control achieved as most of the acute phase of the illness has resolved, and BG has stabilised.

Another study conducted in Brazil by Moreira et al. (13) reported the percentage of patient-days (mean of grouped BG by calendar day for each patient) with all BG values within a predefined optimal range of 4.4 mmol/L– 7.8 mmol/L was only 11.8% in general wards. This study involved patients with diabetes mellitus from 24 hospitals from all regions in Brazil, with almost two-thirds of the participants being 60 years old and above. The author concluded that the most likely reason for the findings was the use of sliding-scale insulin in more than half of patients, which resulted in fluctuating BG. Besides, only 12.9% of patients were seen by diabetes specialists. Our study demonstrated a better percentage of controlled BG as we included patients with SH and none of our patients received sliding-scale insulin.

In several studies, the average LOS for inpatient hyperglycaemia was around 6 days (6, 9). It was longer than in our study (3 days) because those studies included patients in other disciplines, including ICU, where longer LOS is expected. When comparing LOS between the glycaemic control group, Ables et al. (10) found out that LOS for the uncontrolled group was longer (6.17 versus 5.86) days and although significant, may seem trivial (0.31 days, *P* < 0.0001). Though we would expect patients with hyperglycaemia, especially those with uncontrolled BG, to have prolonged hospital stays for their glucose management, the average LOS in our study was considerably short, and we did not find any significant difference between the two groups. This is because, in our practice, patients with uncontrolled BG were attended to by diabetes educators during their hospital stay and were subsequently reviewed early in an endocrine clinic after their discharge. The length of a patient’s stay in the ward would be minimised, effectively preventing hospital-acquired infections that can have severe consequences for diabetes patients. Implementing this measure would also address our hospital’s high bed occupancy rates.

We reported that the percentage of death was higher in the uncontrolled group than in the controlled group, although statistically insignificant. This was consistent with Ables et al. (10), who also found that patients with controlled BG during hospitalisation had lower mortality rates than uncontrolled BG. However, the study found it statistically significant. Our study exhibited a lower overall mortality rate than Moreira et al. (13) (2.5% versus 4.8%), which included patients in the ICU. In contrast, our study specifically excluded patients requiring intensive care.

Our study showed the percentage of readmission within 30 days was higher in the uncontrolled group (7.5% versus 4.6%) than in the controlled group; however, it was statistically not significant (*P* = 0.082). Ables et al. (10) found that readmission rates within 30 days were significantly higher in the uncontrolled group than in the controlled group (16.6% versus 14.1%). Although not mentioned, we can postulate the noticeably higher percentage of readmission was probably due to exacerbations of the most frequent diagnoses and severity of illness, which were chronic obstructive pulmonary disease and cardiac failure, with readmission rates ranging between 17% and 22% and there was no difference between both groups. Both studies have yet to examine the risk factors for readmission and to determine if poorly controlled BG after discharge affects readmissions. To prevent readmission, we would like to suggest patients with uncontrolled BG be co-managed with an endocrine team as Bansal et al. (17), in their study, suggested a significant reduction in 30-day readmission rate following management of diabetes in non-critical medical units by specialised diabetes compared to management by the primary service team.

As evidenced by our study, the high prevalence of inpatient hyperglycaemia calls for heightened awareness among clinicians about the detection and management of inpatient hyperglycaemia through continuous education and training to ensure good glycaemic control in the wards. The limitations of our study include insufficient data on blood investigation. FBS was carried out in 52.2% of T2DM patients and 45.9% of patients with SH. HbA1c was carried out in 57.6% of T2DM patients and 6.8% of patients with SH. Despite limited data, the results showed appropriate findings. Another limitation of this study is that the majority were Malays. We do not look into whether comorbidities, worsening renal function, and risk of hypoglycaemia among our study patients could alter their BG target during admission, albeit hypoglycaemia and advanced CKD were excluded from this study. Further study is needed to examine the management of inpatient hyperglycaemia and include multi-ethnic inclusion with a bigger sample to represent the population of Malaysia.

## Conclusion

The prevalence of inpatient hyperglyacemia was high, with most of the patients having T2DM. More than half of them had uncontrolled BG. Both groups had similar average LOS. A 30-day readmission rate and death during admission were higher in the uncontrolled group, although statistically not significant.

## Figures and Tables

**Table 1:** Demography of patients in controlled group and uncontrolled group

Variables	Overall group (*N* = 841)	Controlled group (*n* = 389)	Uncontrolled group (*n* = 452)	*P-*value
Age (years old)^a^	60 (13.8)	62 (14.5)	59 (13.0)	0.009
Gender, *n* (%)				
Male	453 (53.9)	206 (53.0)	247 (54.6)	0.624^v^
Female	388 (46.1)	183 (47.0)	205 (45.4)	
Race, *n* (%)				
Malay	668 (79.4)	310 (79.7)	358 (79.2)	
Chinese	68 (8.1)	39 (10.0)	29 (6.4)	0.089^v^
Indian	92 (10.9)	36 (9.3)	56 (12.4)	
Others	13 (1.5)	4 (1.0)	9 (2.0)	
Reason for admission, *n* (%)*				
Infections	425 (50.5)	188 (48.3)	237 (52.4)	0.235^v^
CVD	290 (34.5)	135 (34.7)	155 (34.3)	0.900^v^
Nephrology	125 (14.9)	40 (10.3)	85 (18.8)	0.001^v^
Neurology	100 (11.9)	54 (13.9)	46 (10.2)	0.098^v^
Respiratory	62 (7.4)	29 (7.5)	33 (7.3)	0.932^v^
Others	154 (18.3)	64 (16.5)	90 (19.9)	0.196^v^
Hyperglycaemia’s type, *n* (%)				
T2DM	634 (75.4)	212 (54.5)	422 (93.4)	< 0.001^v^
SH	207 (24.6)	177 (45.5)	30 (6.6)	

Notes:

a mean (SD), tested using independent *t*-test;

v chi-square test;

* one patient might have more than one reason for admission

**Table 2:** LOS, FBS, HbA1c, outcome and inpatient treatment of patients in controlled group and uncontrolled group

Variables	Overall group (*N* = 841)	Controlled group (*n* = 389)	Uncontrolled group (*n* = 452)	*P-*value
LOS (in days)^e^	3 (2)	3 (2)	3 (2)	0.347
FBS (mmol/L)^e^	8.0 (4.80) (*N* = 426)	6.6 (2.20) (*n* = 195)	10.2 (6.40) (*n* = 231)	< 0.001
HbA1c (%)^e^	8.0 (3.50) (*N* = 379)	7.0 (1.40) (*n* = 124)	9.2 (3.80) (*n* = 255)	< 0.001
Outcome, *n* (%)				
Discharged home	768 (91.3)	365 (93.8)	403 (89.2)	0.016^v^
Death during admission	21 (2.5)	6 (1.6)	15 (3.3)	0.100^v^
30 day’s readmissions	52 (6.2)	18 (4.6)	34 (7.5)	0.082^v^
Inpatient treatment, *n* (%)				
OAD	184 (21.9)	59 (15.2)	125 (27.7)	< 0.001^v^
Insulin injection	462 (54.9)	88 (22.6)	374 (82.7)	< 0.001^v^
Insulin infusion	32 (3.8)	1 (0.3)	31 (6.9)	< 0.001^v^
No treatment	318 (37.8)	259 (66.6)	59 (13.1)	< 0.001^v^

Notes:

e median (IR) tested using Mann-Whitney U test;

v chi-square test;

* one patient might have more than one inpatient treatment
